# Bio-Inspired Design of Mechanical Properties of Hybrid Topological Cellular Honeycomb Structures

**DOI:** 10.3390/biomimetics10080528

**Published:** 2025-08-12

**Authors:** Yingqiu Sun, Fan Guo, Yangyang Liu

**Affiliations:** 1 School of Electrical Engineering, Chuzhou Polytechnic, Chuzhou 239000, China; 2 School of Engineering, Anhui Agricultural University, Hefei 230036, China; 3 School of Mechanical Engineering, Anhui University of Technology, Ma’anshan 243032, China

**Keywords:** honeycomb structures, bionics design, mechanical property, 3D printing, energy absorption

## Abstract

Inspired by the evolutionary optimization of biological load-bearing systems, honeycomb structures are highly valued in applications involving impact protection and lightweight load-bearing due to their outstanding mechanical properties. This study introduces an interesting honeycomb structure known as the hybrid topological cellular honeycomb structure (HTCHS), which integrates four distinctive topological cells. To effectively fabricate HTCHS samples, the research utilized a fused deposition modeling (FDM) process, employing polyethylene terephthalate glycol-modified (PETG) as the matrix material, successfully producing the HTCHS samples. A finite element simulation model for the HTCHS is created using LS-DYNA software(LS-DYNA R11.1.0 software), and its accuracy is confirmed through a comparative analysis of experimental and simulation results. The influence of the topological cell parameters (*T*_1_ to *T*_4_) on compressive energy absorption, specific energy absorption, and peak crushing force through parametric modeling is investigated. The mechanical properties of honeycomb structures vary depending on the cell parameters at different positions, and monotonically increasing the design parameters does not improve the energy absorption capacity of the HTCHS. To enhance the mechanical performance of the HTCHS, the initial periodic cell configurations are transformed into non-periodic designs. A discrete optimization design framework for local parameters of the HTCHS is established, integrating cell coding with the MOPSO algorithm. The feasibility of the optimization results is validated through experimental data, demonstrating that this study offers an effective technical solution for developing a novel generation of cellular honeycomb structures with customizable mechanical properties.

## 1. Introduction

Impact dynamics, a fundamental mechanical interaction phenomenon, intricately weaves through both advanced engineering systems and the fabric of everyday life. In critical domains such as aerospace [1,2], transportation [3,4,5], and specialized equipment [6,7], the repercussions of impact events can be nothing short of catastrophic, leading to structural failures and complete system breakdowns. This stark reality highlights the urgent need for cutting-edge protective structures boasting exceptional energy dissipation capabilities. Among the vanguard of contemporary energy-absorbing designs, honeycomb structures stand out as particularly compelling candidates. Their remarkable properties have propelled them into widespread use across aerospace, transportation, and biomedical fields. With exceptional specific strength, unparalleled energy absorption capacity, and adaptable deformation mechanisms, honeycomb structures represent a fusion of innovation and practicality, poised to redefine safety and resilience in engineering applications [8,9,10,11,12].

The excellent mechanical performance design of honeycomb structures initially originated from the imitation and reference of efficient structural and mechanical optimization of natural biomaterials such as bee hives, bones, and plant cells. Traditional honeycomb configurations predominantly employ elementary geometric forms such as hexagonal, triangular, and circular cellular structures. The analysis of the mechanical properties of different honeycomb structures under small-scale deformation using elastic mechanics methods has been reported [13,14,15,16]. However, cellular honeycomb structures mainly exhibit large-scale deformation mechanical behavior during crushing protection. Recent years have witnessed significant advancements in crushing protection through geometric optimization. Deng et al. [17] pioneered a triangular gradient rib honeycomb demonstrating a 63.84% improvement in specific energy absorption over conventional gradient designs, while simultaneously establishing optimal bottom-layer angle parameters. Parallel innovations in manufacturing have emerged, exemplified by Xu et al.’s [18] vacuum sintering technique for aluminum honeycombs, which achieves superior metallurgical bonding and exhibits characteristic X-shaped shear deformation modes. Biomimetic approaches have proven particularly fruitful, with San Ha et al. [19] developing hierarchical circular honeycombs inspired by natural structures that achieve 45.3–71.2% greater energy absorption than conventional designs. The velocity-dependent behavior of these structures has been thoroughly characterized by Ren et al. [20], who elucidated the critical role of negative Poisson’s ratio effects in low-velocity impact scenarios. Through quasi-static and dynamic compression tests, Wang et al. [21] revealed anisotropic collapse behavior in square honeycomb cores and developed empirical formulas to characterize high-velocity compression responses and energy absorption. Inspired by square porous wood and DNA double helix, Zhao et al. [22] designed novel helical layered square honeycombs that significantly enhance mechanical performance by leveraging helical layers to mitigate stress concentration, promote uniform ‘X’-shaped deformation bands, and shift failure mechanisms from fiber tearing to controlled layer cracking. Inspired by bamboo microstructure and bat wing morphology, Xu et al. [23] developed two novel nested gradient circular honeycombs that demonstrate better energy absorption performance compared to regular honeycombs through a unique nested gradient strategy that optimizes deformation modes across different loading rates while outperforming existing gradient honeycomb designs. Although the mechanical properties and energy absorption capacity of honeycomb structures have been significantly improved through typical geometric cross-section optimization and biomimetic design strategies, the basic configuration limitations of typical honeycomb cells still constrain their breakthrough development.

Recent years have witnessed the emergence of innovative topological designs for honeycomb cells to overcome the limitations of conventional geometric configurations. These advanced structures demonstrate remarkable improvements in energy absorption characteristics, dynamic impact response, and multifunctional integration through strategic cell configuration. A novel Diabolo-shaped honeycomb structure exhibited 22.7~141.3% higher plateau stress and 7.3–103.7% greater specific energy absorption compared to traditional re-entrant hexagonal honeycombs across various impact velocities [24]. Significant progress has been made in 3D-printed bi-material multistable auxetic honeycombs, achieving tunable Poisson’s ratio and multi-stage stress plateaus for enhanced energy absorption, with comprehensive investigations of deformation mechanisms and performance dependencies on geometric parameters and crushing velocity [25]. The development of self-similar inclusion-enhanced auxetic honeycombs has led to substantially improved mechanical performance, identifying critical geometric parameters for optimal energy absorption and buckling resistance in protective applications [26]. Particularly noteworthy is the re-entrant diamond-enhanced honeycomb design that synergistically combines geometric optimization with bi-material architecture, overcoming the traditional trade-off between energy absorption and auxetic effects. This configuration achieved a 23.7% increase in specific energy absorption (1.20 J/g) while enhancing the negative Poisson’s ratio by 50% (from −0.36 to −0.54) through precisely controlled material distribution and geometric parameters [27]. These advancements collectively demonstrate that optimized geometric configurations combined with multi-material integration can significantly enhance energy absorption, mechanical tunability, and auxetic performance.

The development of hybrid-cell honeycombs has further expanded the design space for lightweight, high-performance structures through the strategic combination of diverse cellular geometries. A star-rhombic honeycomb configuration demonstrated 136% and 75% higher specific energy absorption than conventional reentrant and star honeycombs, respectively, while maintaining excellent auxetic properties [28]. The integration of octagonal and hexagonal unit cells in a re-entrant hybrid honeycomb structure resulted in superior negative Poisson’s ratio performance and enhanced deformation capability compared to conventional designs [29]. Considerable progress has been made in overcoming the stiffness-energy absorption trade-off through hybrid honeycomb structures combining hexagonal and re-entrant configurations, with dual-material (PLA/TPU) variants showing exceptional repeatable loading performance in quasi-static compression tests [30]. Novel auxetic hybrid structures integrating re-entrant cells with face-centered cubic cells have significantly improved deformation stability and energy absorption under multi-axial loading, effectively addressing the traditional re-entrant honeycomb’s limitations in lateral stiffness, as confirmed through comprehensive numerical and experimental analyses of geometric parameter effects [31]. The application of inverse design frameworks combining neural network prediction with active learning optimization has enabled the creation of lightweight hybrid honeycomb structures with superior impact resistance, achieving 94.98% energy absorption while simultaneously reducing peak stress by 28.85% and mass by 19.91% [32]. A star double-arrow honeycomb design featuring synergistic integration of star and double-arrow geometries demonstrated unique two-stage deformation behavior with independently tunable second plateau stress (controlled through double-arrow parameters) and enhanced energy absorption capacity, as validated through theoretical modeling, numerical simulation, and experimental investigations that identified star cell-wall angles as primary determinants of deformation modes and auxetic properties [33]. These developments conclusively demonstrate that the strategic combination of classical topological cells into novel hybrid configurations can substantially enhance the mechanical performance of honeycomb structures.

To further expand the design and application of hybrid topology cells, hybrid topological cellular honeycomb structures are designed in this study. This design method is based on the combination of four classic honeycomb cells, and all basic cells have the same connection point. Based on this connection point, honeycomb cells can be arranged in a regular array in the horizontal plane, ultimately forming a novel hybrid topological cellular honeycomb structure. The mechanical properties of hybrid topological cellular honeycomb structures are investigated through a combination of experimental testing and numerical simulation. To enhance the mechanical performance of the HTCHS, the initial periodic cell configurations are transformed into non-periodic designs. A discrete optimization design framework for local parameters of the HTCHS is also established in this study.

## 2. Design and Experiments

**Figure 1:**
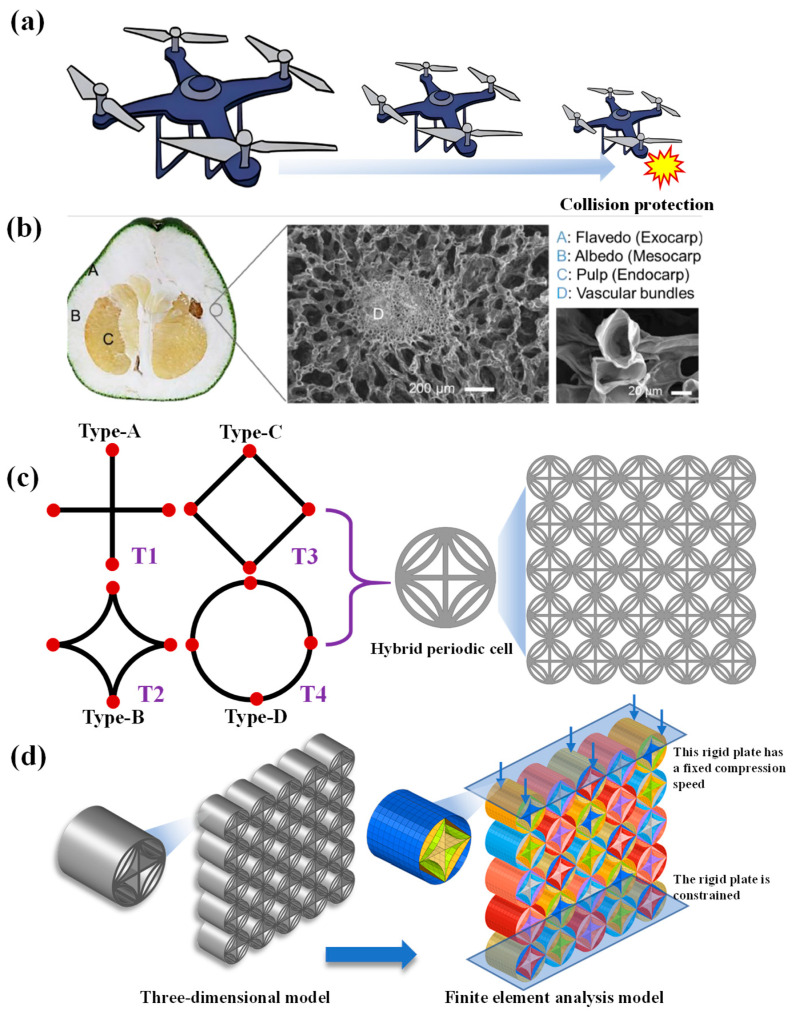
Design scheme (**a**) A Collision case; (**b**) Biomaterials [34]; (**c**) Structural design schemes; (**d**) Finite element models.

Taking aircraft structural design as an example, complex and changing environmental factors can easily cause unexpected impacts between the aircraft and the ground, as shown in Figure 1a. Such impacts may not only cause structural damage to the aircraft but also trigger secondary disasters such as fires. Therefore, the design of aircraft buffer structures needs to achieve efficient energy absorption mechanisms to reduce impact loads, employ controllable deformation characteristics to achieve orderly energy dissipation, adopt lightweight design to balance protective performance and flight efficiency, effectively protect key components in uncertain impact environments, suppress the chain development of disasters, and ultimately ensure the operational safety and feasibility of the aircraft. Biomaterials offer exceptional inspiration for designing multicellular structures with superior mechanical properties. As illustrated in Figure 1b [34], the pomelo peel demonstrates this principle through its composite structure consisting of flavedo and spongy albedo containing vascular bundles embedded in loose tissue. Previous studies investigating hydration state, density, and vascular bundle arrangement have revealed this natural material’s hierarchical energy-absorption mechanism, providing valuable insights for engineering applications [35]. X-ray tomography analysis further demonstrates how the interconnected vascular-cell networks significantly enhance impact resistance [36]. These different microscopic cellular structures effectively enable biological systems to withstand external loads. Based on these biomimetic principles, this study proposes a hybrid topological cellular honeycomb structure (HTCHS) by utilizing the characteristic of different adjacent cells in the honeycomb structure having the same connection points. The honeycomb structure is constructed by continuously expanding the cells, and its specific configuration is shown in Figure 1c. The HTCHS integrates multiple topological features, mainly including four types of topological cells, namely type A to type D. Type A is a cross-shaped cross-section, type B is an arc-shaped cross-section, type C is a square cross-section, and type D is a circular cross-section, all of which have common periodic connection points. The aim is to fully leverage the advantages of different topological structures to enhance the overall mechanical performance of honeycomb structures.

To evaluate the mechanical protection performance of the proposed HTCHS, a 5 × 5 cell array is selected as the research object, as shown in Figure 1d. An explicit dynamic compression simulation model is established on the LS-DYNA platform, which is widely used in dynamic mechanical simulations. To ensure consistency among different topological structures, the circumscribed circle diameter of all topological cells in the model is uniformly set to 10 mm. Additionally, to further investigate the influence of topological cell thickness on the mechanical properties of the honeycomb structure, *T*_1_ to *T*_4_ are used to represent different cell thicknesses for parametric analysis. The simulation model primarily consists of the honeycomb structure and upper/lower plates. For boundary conditions, the lower plate is fixed to simulate a rigid support, while the upper plate is compressed at a constant velocity to replicate external loading conditions. Both plates are modeled using rigid elements. The material behavior of the honeycomb structure is simulated using the MAT24 model in LS-DYNA, which accurately captures the elastoplastic response under compressive loading.

**Figure 2:**
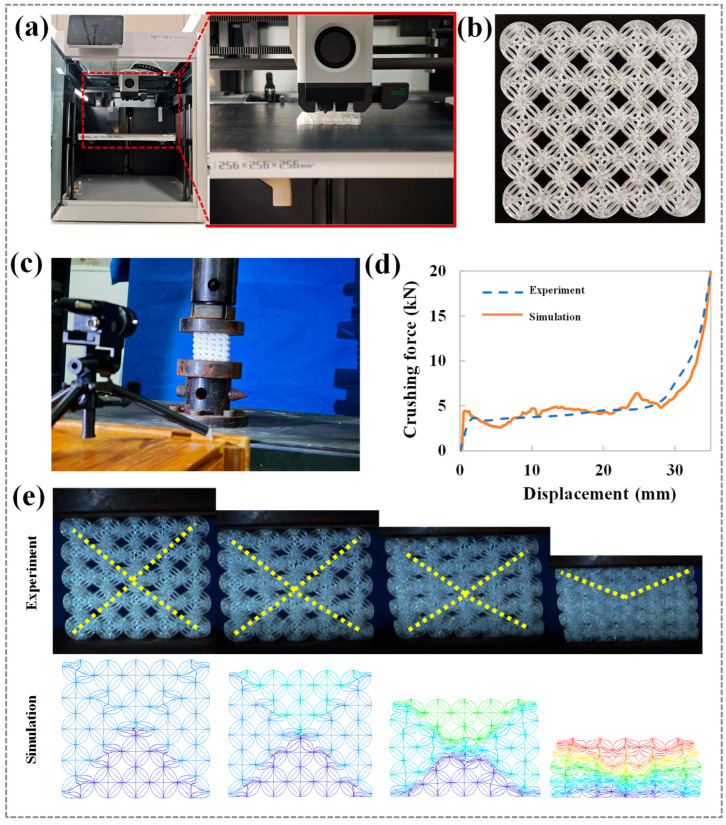
Experimental results (**a**) 3D printing; (**b**) Test specimen; (**c**) Compression test; (**d**) Compression force curves; (**e**) Deformation modes.

During finite element mesh generation, this study conducted multiple simulation tests to balance computational efficiency and accuracy. Through iterative adjustment of mesh sizes and comparison of result convergence and accuracy, an optimal finite element mesh size of 1 mm is determined. This resolution ensures sufficient accuracy in reflecting the honeycomb structure’s mechanical properties and deformation characteristics while maintaining reasonable computation times, thereby improving research efficiency. The polyethylene terephthalate glycol-modified (PETG) as the matrix material is selected due to its excellent toughness, favorable mechanical properties (including high strength and toughness), and good processability for additive manufacturing. The key material parameters include: density = 1.14 × 10^3^ kg/m^3^, Poisson’s ratio = 0.32, Young’s modulus = 1.28 GPa, and yield strength = 50 MPa. As shown in Figure 2a, using fused deposition modeling (FDM) technology (Bambu Lab X1 machine), HTCHS samples are successfully fabricated. Through precise control and optimization of printing parameters, the manufactured specimens exhibited good formability, surface quality, and absence of visible defects (e.g., cracks or pores), providing reliable samples for subsequent mechanical testing, as shown in Figure 2b.

To verify the accuracy of the simulation model, compression tests are conducted on HTCHS specimens using a universal testing machine (CMT5105 electronic universal testing machine, SASTest, Shenzhen, China), as shown in Figure 2c. During testing, a compressive load is applied to the specimens at a constant speed of 2 mm/min, and the load–displacement curves are recorded. The experimentally obtained compressive force curve is compared with simulation results, as shown in Figure 2d. The results show that the variation trends of the compressive force curves in the experiment and simulation are highly consistent. Despite some fluctuations in force values, both methods accurately reflect the compressive force-displacement relationship. This comparison preliminarily verifies the effectiveness of the simulation model in representing the compressive mechanical behavior of HTCHS. As shown in Figure 2e, further analysis of HTCHS deformation under in-plane compression reveals that, similar to traditional single-cell honeycomb structures, HTCHS forms distinct shear band regions during compression [37]. The formation of shear bands represents an important energy dissipation mechanism and deformation localization characteristic in honeycombs under compressive loads, with their formation depending on the topological structure and material properties. Comparison between simulated and experimental deformation modes shows good consistency, further confirming the feasibility of the simulation modeling approach. This method not only accurately predicts HTCHS mechanical properties and deformation modes but also provides an effective tool for parametric analysis and optimization design.

## 3. Parameter Analysis

Honeycombs, as a typical cellular material structure, mainly rely on their ability to absorb external energy. This energy dissipation mechanism effectively alleviates the mechanical damage suffered by the protected object. In this research work, three key indicators, energy absorption (*EA*) [12,38], specific energy absorption (*SEA*) [39], and peak crushing force (*PCF*) [40], are selected to evaluate the mechanical protection performance of HTCHS. Among them, EA represents the total energy absorbed during the effective compression process and can be calculated as

(1) $$EA=\int_{0}^{D_{d}}F(x)dx$$

where F(x) denotes the instantaneous compression force. $D_{d}$ is the compression displacement. SEA represents the energy absorbed per unit mass, which is a key indicator for determining the absorption capability and can be calculated as

(2) $$SEA=\frac{\int_{0}^{S_{d}}F(x)dx}{Mass}$$

Here, Mass denotes the total weight of the HTCHS. In addition, PCF represents the initial peak load per unit area at the end of the honeycomb.

**Figure 3:**
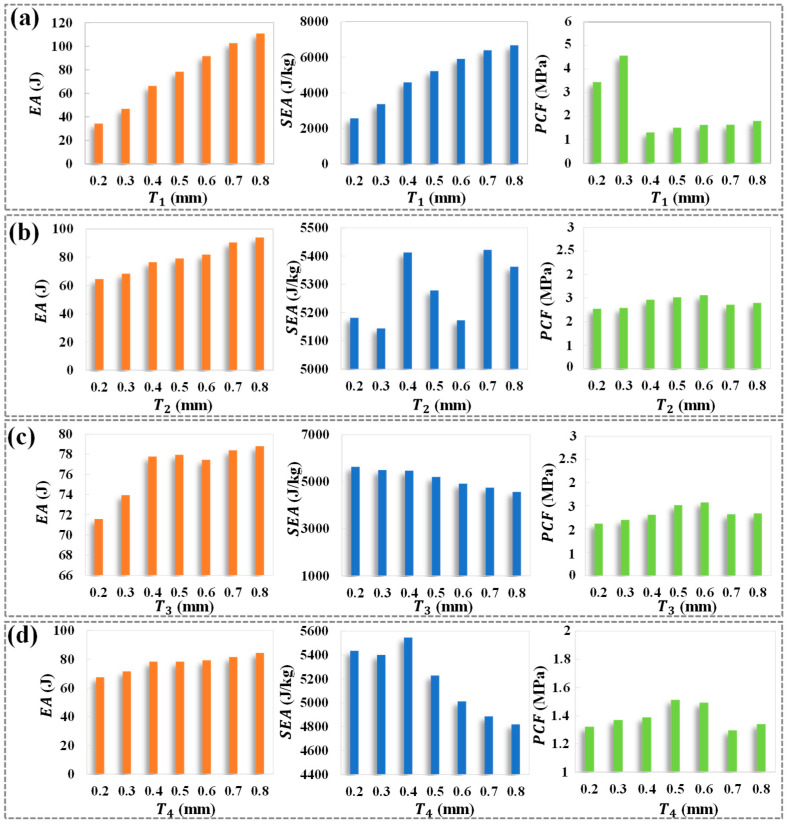
Energy absorption characteristics with a single thickness change (**a**) The influence of *T*_1_; (**b**) The influence of *T*_2_; (**c**) The influence of *T*_3_; (**d**) The influence of *T*_4_.

Figure 3 illustrates the influence of different design parameters (*T*_1_, *T*_2_, *T*_3_, *T*_4_) on the energy absorption-related indicators of HTCHS. As shown in Figure 3a, when *T*_1_ varies, both the *EA* and *SEA* show a significant positive increasing trend, with their patterns being relatively similar. However, after reaching a maximum value at approximately *T*_1_ = 0.3 mm, the *PCF* remains stable within the range of *T*_1_ = 0.4~0.8 mm, indicating that changes in *T*_1_ have a limited effect on the maximum compressive impact force sustained by HTCHS. In Figure 3b, as the design parameter *T*_2_ increases, the *EA* continues to rise significantly, though *SEA* exhibits a distinct nonlinear response: it first increases, then decreases, and increases again. The highest *SEA* value is achieved at *T*_2_ = 0.7 mm. Meanwhile, the *PCF* initially increases with *T*_2_ and then slowly decreases, with the lowest and highest *PCF* values occurring at *T*_2_ = 0.2 mm and *T*_2_ = 0.6 mm, respectively. This suggests that *T*_2_ has a nonlinear influence on both the energy absorption efficiency and peak compression force of HTCHS. As shown in Figure 3c, although *EA* shows a slight increasing trend with *T*_3_ increasing, the variation is minimal within the range of *T*_3_ = 0.4~0.8 mm. Notably, the *SEA* decreases as *T*_3_ increases, primarily due to the increase in material weight, which does not enhance the energy absorption capacity of HTCHS but reduces efficiency per unit mass. Additionally, *PCF* displays a trend of first increasing and then decreasing within the range of *T*_3_ = 0.2~0.8 mm, with its maximum at *T*_3_ = 0.6 mm. In terms of *T*_4_, *EA* shows a slight increasing trend as *T*_4_ increases (in Figure 3d). The *SEA* exhibits a behavior similar to *T*_2_, increasing initially and then decreasing, reaching its peak at *T*_4_ = 0.4 mm. The *PCF* for HTCHS also shows a nonlinear trend, with its maximum value occurring at *T*_4_ = 0.5 mm. The mechanical properties of honeycomb structures vary depending on the cell parameters at different positions, and monotonically increasing the design parameters does not improve the energy absorption capacity of the HTCHS.

**Figure 4:**
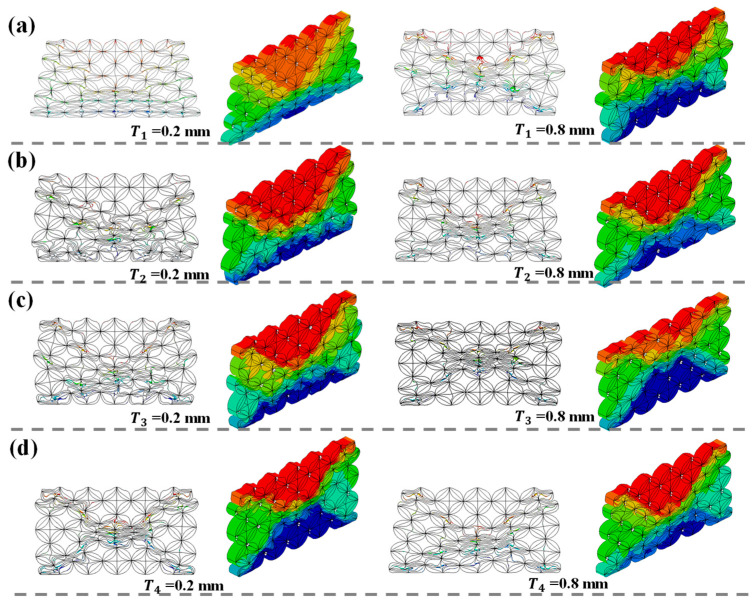
Deformation modes (**a**) Different deformation modes of *T*_1_; (**b**) Different deformation modes of *T*_2_; (**c**) Different deformation modes of *T*_3_; (**d**) Different deformation modes of *T*_4_.

To gain a deeper understanding of the mechanical properties of HTCHS under different thickness parameters, Figure 4 illustrates the deformation modes of HTCHS when different positional parameters are altered. All HTCHS samples exhibit pronounced shear band characteristics during compression. However, there are differences in the shear band variation modes before and after altering different positional parameters. For instance, when *T*_1_ = 0.2 mm, HTCHS primarily demonstrates a deformation mode characterized by end collapse. Conversely, when *T*_1_ = 0.8 mm, HTCHS mainly exhibits a deformation mode with an “X”-shaped shear band. For parameter variations in *T*_2_ to *T*_4_, HTCHS mainly displays an “X”-shaped shear band deformation mode. Theoretically, as the material dosage increases, HTCHS will enhance its resistance to deformation to a certain extent, leading to an increase in *PCF*. Nevertheless, the increase in different positional parameters also alters the deformation-triggering mode of HTCHS, thereby affecting the peak load during initial compression. This phenomenon explains the interesting trend shown in Figure 3, that is, within a specific design space, as the design parameters increase, *PCF* shows a downward trend.

**Figure 5:**
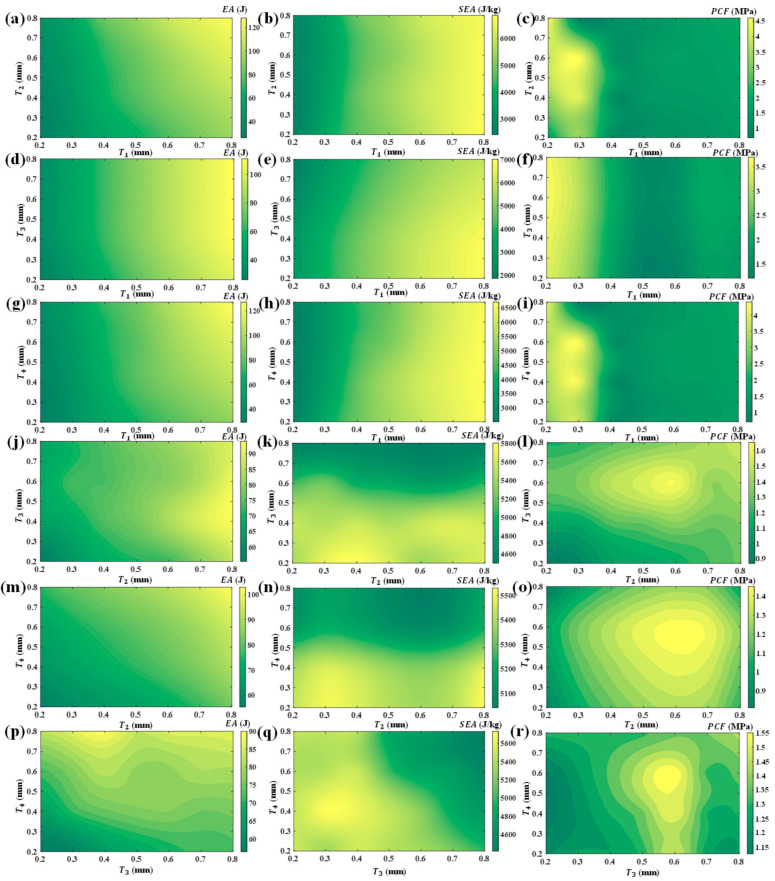
Energy absorption characteristics under various thickness changes (**a**) *EA* of *T*_1_ and *T*_2_ changes; (**b**) *SEA* of *T*_1_ and *T*_2_ changes; (**c**) *PCF* of *T*_1_ and *T*_2_ changes; (**d**) *EA* of *T*_1_ and *T*_3_ changes; (**e**) *SEA* of *T*_1_ and *T*_3_ changes; (**f**) *PCF* of *T*_1_ and *T*_3_ changes; (**g**) *EA* of *T*_1_ and *T*_4_ changes; (**h**) *SEA* of *T*_1_ and *T*_4_ changes; (**i**) *PCF* of *T*_1_ and *T*_4_ changes; (**j**) *EA* of *T*_2_ and *T*_3_ changes; (**k**) *SEA* of *T*_2_ and *T*_3_ changes; (**l**) *PCF* of *T*_2_ and *T*_3_ changes; (**m**) *EA* of *T*_2_ and *T*_4_ changes; (**n**) *SEA* of *T*_2_ and *T*_4_ changes; (**o**) *PCF* of *T*_2_ and *T*_4_ changes; (**p**) *EA* of *T*_3_ and *T*_4_ changes; (**q**) *SEA* of *T*_3_ and *T*_4_ changes; (**r**) *PCF* of *T*_3_ and *T*_4_ changes.

It is important to recognize that the various local position parameters do not act in isolation when influencing the mechanical properties of HTCHS. As illustrated in Figure 5, when both *T*_1_ and *T*_2_ increase simultaneously, the energy absorption of HTCHS shows a pronounced upward trend, with higher *EA* values readily achievable at the periphery of the design space. This intriguing pattern is similarly observed when other design parameters are varied in combination. Conversely, when *T*_2_, *T*_3_, and *T*_4_ are increased together, the *PCF* of HTCHS reveals remarkable nonlinear characteristics, with its maximum value occurring near the center of the design space. When *T*_1_ remains unchanged, simultaneous changes in *T*_2_, *T*_3_, and *T*_4_ may result in a smaller *PCF* at the boundary positions of the design parameter space. This provides an opportunity to simultaneously regulate higher *SEA* and lower *PCF*. Notably, within this realm, the specific energy absorption can surpass an impressive 7000 J/kg. Exploring the synergistic interactions among these design parameters and their influence on mechanical properties provides insights for precisely modulating HTCHS’s characteristics and enhancing its performance in impact protection.

## 4. Performance Customization

Recent research compellingly illustrates that reimagining the traditional periodic arrangement of cells in a honeycomb structure into a non-periodic configuration can significantly enhance its mechanical properties, including energy absorption, load distribution, and damage tolerance [41,42,43]. This innovative transformation disrupts the conventional symmetry and uniformity associated with periodic structures, introducing controlled geometric disorder that alters stress propagation and deformation mechanisms. As a result, non-periodic honeycombs exhibit a remarkable diversity in mechanical responses, including sequential buckling, localized collapse, and graded stiffness. By integrating additional structural variations, such as graded cell sizes, hybrid wall thicknesses, or hierarchical substructures, a non-periodic honeycomb can exhibit an expansive range of tunable mechanical behaviors when subjected to compressive, shear, or impact forces. Computational and experimental studies confirm that this adaptability enables precise fine-tuning of stiffness, strength, and energy dissipation characteristics. Consequently, such structures demonstrate exceptional performance tailored for specific applications, including lightweight aerospace components, impact-resistant armor, and energy-absorbing automotive structures, where traditional periodic designs fall short.

**Figure 6:**
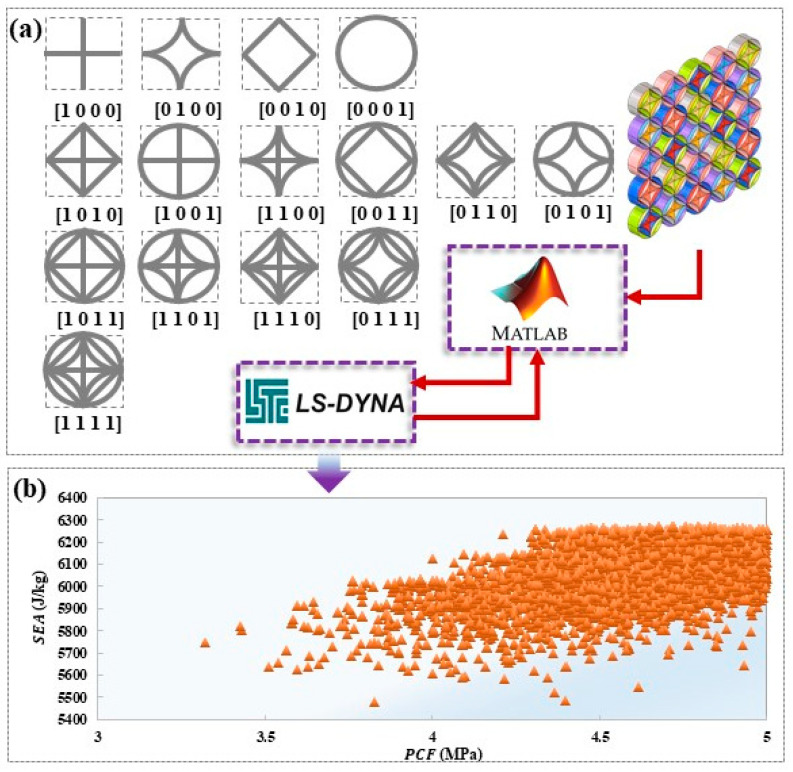
Design parameters and optimization results (**a**) Structural design coding; (**b**) Optimization results.

This study leverages the research approach of reimagining honeycomb structures by conducting parametric design and optimization using a 5 × 5 cell array as a case study. In the context of in-plane compression, the types of cells situated at distinct locations within the HTCHS are pivotal to the overall mechanical response. To streamline the subsequent analysis and calculations, each cell type at various positions is defined as an independent design parameter. This study incorporates coding to delineate different cell types and their presence or absence, with specific coding rules illustrated in Figure 6a. As a result of this methodology, the cells positioned in diverse local areas of the HTCHS can generate 15 hybrid forms through a variety of combinations.

**Figure 7:**
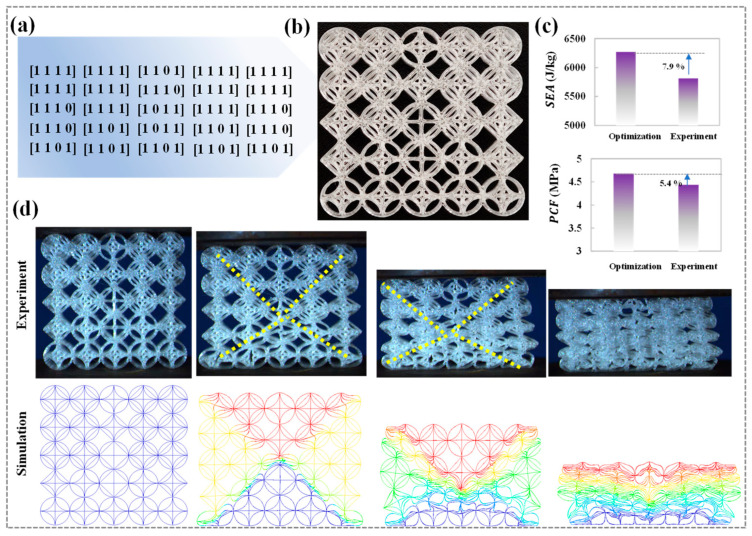
Optimization results and validation (**a**) The optimization result; (**b**) The optimized design scheme; (**c**) Error analysis; (**d**) Deformation modes.

In optimization problems that involve high-dimensional design parameters, heuristic optimization algorithms offer significant advantages. This research employs the classic multi-objective particle swarm optimization (MOPSO) algorithm to perform the optimization calculation [44,45]. The optimization process is implemented using an automated coupling framework. Firstly, the design variable parameters are dynamically configured through the MATLAB (R2023a) script interface, and the parameter set is transmitted in real-time to the LS-DYNA solver for explicit dynamic calculations. After the simulation is completed, the MATLAB post-processing module automatically parses the result file and extracts the objective function value to drive the multi-objective optimization algorithm for iterative optimization, as shown in Figure 6a. Within this optimization framework, the cell parameters at various local positions are regarded as independent discrete variables. The particle swarm iteratively navigates the solution space to determine the optimal combination of parameters that fulfills multiple optimization objectives. To assess the best configurations for different cell combinations, a uniform wall thickness of 0.8 mm is established. This choice aims to limit the effects of thin wall thickness on the optimization results, allowing for a more focused optimization process concerning the different cell type combinations. The optimization objective is defined as maximizing *SEA* and minimizing *PCF*. Through MOPSO calculations, a Pareto solution set is generated, showcasing various feasible solutions that illustrate the trade-offs between the different optimization objectives, as shown in Figure 6b. To refine this solution set, the minimum distance method is utilized to select a group of optimal solutions, with their specific parameters and performance characteristics detailed in Figure 7a.

To verify the feasibility and accuracy of the optimization results, this study utilized fused deposition modeling technology to recreate samples according to the optimized scheme, as illustrated in Figure 7b. A comparative analysis is performed between the optimized outcomes and experimental results. The findings revealed that the maximum error between the optimized results and experiments is maintained within 8% (Figure 7c), underscoring the efficacy of the optimization algorithm. Additionally, the deformation modes observed in both experiments and simulations are notably consistent, characterized by intriguing left-right symmetrical shear band deformation modes (Figure 7d). This deformation mode further supports the validity of the optimized scheme in terms of mechanical properties, suggesting that the proposed non-periodic cell design optimization approach can effectively influence the mechanical behavior of the HTCHS, thus providing valuable design strategies for their application in engineering protection. This study established an optimization framework by encoding topological cells, which can provide a design method for non-periodic honeycomb designs with different mechanical performance requirements. In addition, for different topology cell types or large-scale mechanical structural components, the design optimization idea of non-periodic topological honeycomb cells can be used to customize their mechanical properties.

This study introduces a non-periodic hybrid topology cellular structure, which is suitable for scalable engineering applications. Although the current prototype is made of simple plastic, this material selection does not limit its potential for cross-scale adaptation or material generalization. Specifically, in the field of engineering protection, the design method can be applied to impact mitigation for precision machinery (such as drones and transportation packaging), personal safety protection (such as helmets), and vehicle collision safety protection (e.g., energy-absorbing components). Compared to traditional periodic honeycomb structures, non-periodic designs can more flexibly adjust the distribution of materials under complex loads, reduce fluctuations in energy transfer processes, and are particularly suitable for scenarios that require a balance between lightweight and impact resistance. Despite the high complexity of their manufacturing processes, the maturity of additive manufacturing technologies, such as 3D printing, has made the implementation of non-periodic honeycomb cell combination designs feasible.

## 5. Conclusions

This study proposes a hybrid topological cellular honeycomb structure (HTCHS) and investigates its mechanical properties through integrated experimental and computational approaches. The results have demonstrated that localized modifications of design parameters within the HTCHS critically influence its *EA*, *SEA*, and *PCF*. Interestingly, although increasing parameter values elevates total energy absorption, this does not universally improve the structural energy absorption capacity; conversely, *SEA* may exhibit a reduction under certain parametric combinations. The formation mechanism of shear bands before and after changes in design parameters is revealed. For instance, at *T*_1_ = 0.2 mm, the HTCHS predominantly displays end-collapse-induced deformation, whereas a transition to *T*_2_ = 0.8 mm triggers a dominant “X”-shaped shear band mode. Similar “X”-shaped deformation modes are consistently observed for cell parameters *T*_2_~*T*_4_. To harness the tunability of multi-parameter hybrid cells, a multi-objective optimization framework is implemented. The minimum distance method enabled the selection of optimal solutions from the Pareto frontier, with subsequent validation against experimental and finite element analysis data. The optimization results show good consistency, with a maximum relative error of less than 8%, confirming the effectiveness of the algorithm. Although the current prototype of HTCHS is made of PETG plastic, this material selection does not limit its potential for cross-scale adaptation or material generalization. In summary, this work establishes that multi-parametric optimization of hybrid topological cells can precisely regulate HTCHS’s mechanical response, offering a feasible design paradigm for impact-protective structures.

## Data Availability

The original contributions presented in this study are included in the article. Further inquiries can be directed to the corresponding author.
